# Topographic sketching for surgical planning after neoadjuvant chemotherapy in breast cancer: a non-invasive localization method

**DOI:** 10.1007/s00423-026-04016-3

**Published:** 2026-03-18

**Authors:** Mahmut Muslumanoglu, Baran Mollavelioglu, Selman Emiroglu, Hasan Berke Atalay, Neslihan Cabioglu, Ravza Yılmaz, Rana Gunoz Comert, Aysel Bayram, Sidar Bagbudar, Melin Aydan Ahmed, Mustafa Tükenmez

**Affiliations:** 1 Department of General Surgery, Istanbul Faculty of Medicine, Istanbul University, Topkapi, Turgut Ozal Millet Avenue, 34093 Istanbul, Turkey; 2 Department of Radiology, Istanbul Faculty of Medicine, Istanbul University, Istanbul, Turkey; 3 Department of Pathology, Istanbul Faculty of Medicine, Istanbul University, Istanbul, Turkey; 4 Department of Medical Oncology, Institute of Oncology, Istanbul University, Istanbul, Turkey

**Keywords:** Neoafjuvant chemotherapy, Breast-conserving surgery, Localization method, Tumor marking, Metallic marker, Guide wire

## Abstract

**Purpose:**

Neoadjuvant chemotherapy (NAC) is widely adopted as the standard treatment for locally advanced breast cancer. However, following NAC, tumors may become non-palpable due to complete or partial response, making it challenging to achieve adequate margins in breast-conserving surgery (BCS). Various tumor localization techniques have been developed to address this issue. This study compares traditional clip and wire-guided marking (Radiological Marking, RM) with a non-invasive technique called Topographic Sketching (TS).

**Methods:**

This retrospective study included 208 patients who underwent BCS after their tumors became non-palpable post-NAC. A total of 102 patients underwent RM using radiopaque clips followed by wire-guided excision, while 106 patients were treated using the TS technique. In the TS group, pre-NAC imaging and physical examination were used to determine the tumor’s location, distance from anatomical landmarks, and skin projection. These data were documented photographically and archived. Post-NAC, the tumor site was re-evaluated, and the excision was guided by redrawn topographic markings based on both archival and current imaging data.

**Results:**

Among the participants, 91 patients showed complete response and 117 showed partial response to NAC. Unifocal tumors were observed in 168 patients, and multifocal tumors in 40. Only one patient from each group required a second surgery due to positive surgical margins. During a median follow-up of five years, six patients experienced local recurrence. There were no statistically significant differences between the RM and TS groups in terms of patient age, tumor stage, nodal status, multifocality, pathological response, or molecular subtype.

**Conclusion:**

Topographic sketching is a promising, non-invasive, and cost-effective alternative to clip and wire-guided localization. It provides critical information about the tumor’s original borders and is comparably effective in terms of surgical outcomes and recurrence rates.

## Introduction

Neoadjuvant chemotherapy (NAC) has emerged as the standard of care for patients with locally advanced breast cancer in recent years [1, 2]. By enabling significant tumor shrinkage, NAC has expanded the eligibility for breast-conserving surgery (BCS), even in patients initially considered candidates for mastectomy. In addition to increasing breast-conserving surgery rates, neoadjuvant chemotherapy provides valuable information regarding tumor response, which may influence post-operative adjuvant treatment decisions. In cases of axillary lymph node involvement, axillary dissection may be avoided if a pathological complete response (pCR) is achieved post-NAC. Notably, studies have shown no significant difference in survival or local recurrence rates between patients undergoing BCS and those receiving mastectomy. Given its advantages, particularly with regard to cosmetic outcomes, BCS has become the preferred approach for many patients [3].

However, the tumor response to NAC is highly variable. While a complete or partial response is frequently observed, the resulting reduction in tumor volume can pose challenges in accurately identifying the surgical excision margins, potentially increasing the need for re-excision. Achieving negative surgical margins is critical to ensure local disease control, and preoperative localization techniques play a pivotal role in this context.

For tumors rendered non-palpable after NAC, several localization methods have been developed. Among the most widely used is the placement of a radiopaque clip within the tumor bed under ultrasound or mammographic guidance before the initiation of chemotherapy [4]. Even in cases of near-complete or complete pathological response, the marked area can typically be visualized using imaging modalities, facilitating accurate localization with wire-guided excision. Studies have demonstrated that metallic clip placement significantly improves the likelihood of achieving negative margins [5].

Despite their effectiveness, clip and wire-based localization techniques have notable limitations. These include logistical challenges, such as the need for same-day wire placement by a radiologist, as well as potential complications like pain, bleeding, wire migration, and—in rare cases—retained fragments, pneumothorax, or hemothorax [6]. Moreover, these methods are resource-intensive and may not be feasible in all clinical settings.

Other alternative approaches such as radioguided occult lesion localization (ROLL), radioactive seed localization (RSL), magnetic seed localization, radar-based localization systems (Savi Scout^®^), and carbon or skin tattooing have been introduced [7–11]. While these techniques may improve scheduling flexibility and patient comfort, they often require dedicated equipment, additional costs, regulatory approval, or specialized logistical support. Consequently, access to such technologies may be limited in many institutions.

In this study, we aimed to evaluate the clinical utility of an alternative, non-invasive localization technique—Topographic Sketching (TS)—and to compare its outcomes with those of conventional radiological marking methods.

## Methodology

**Fig. 1:**
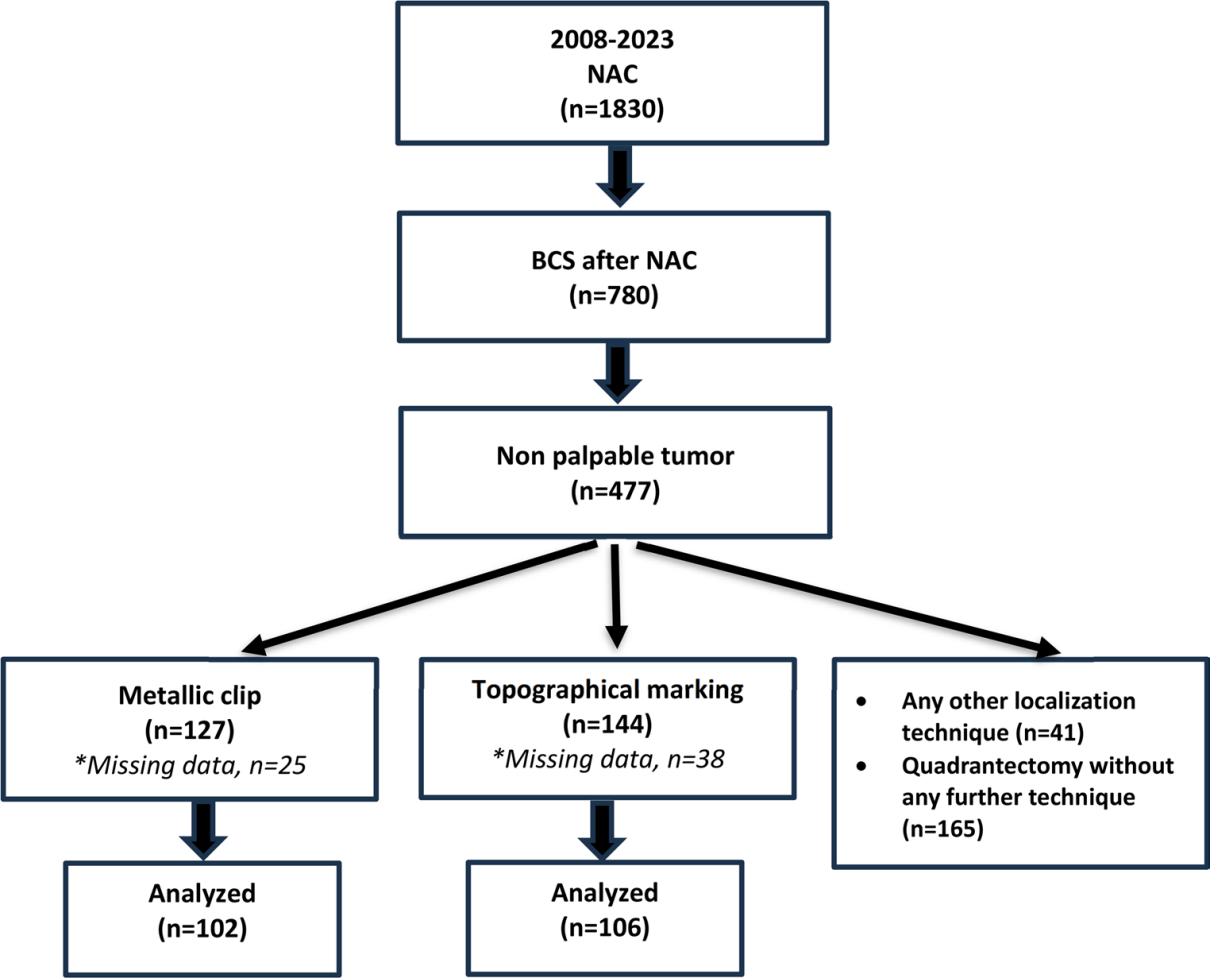
– Flowchart of the study

Between January 1, 2008, and October 31, 2023, a total of 208 patients diagnosed with breast cancer at the Department of General Surgery, Istanbul Faculty of Medicine, were retrospectively included in this study. All patients underwent breast-conserving surgery (BCS) following neoadjuvant chemotherapy (NAC) and had non-palpable tumors at the time of surgery (Fig. 1).

Patients were divided into two groups based on the localization technique used. In 102 patients, radiological marking (RM) was performed using ultrasound-guided radiopaque clip placement before NAC, followed by wire-guided localization on the day of surgery. In the remaining 106 patients, the Topographic Sketching (TS) technique was applied.

In the RM group, bidirectional mammograms were taken after clip placement to confirm correct localization. After NAC, the residual tumor or the clip was visualized using imaging, and the area was marked with a hooked wire under radiological guidance. The presence of the clip in the excised specimen was confirmed with mammography.

**Fig. 2:**
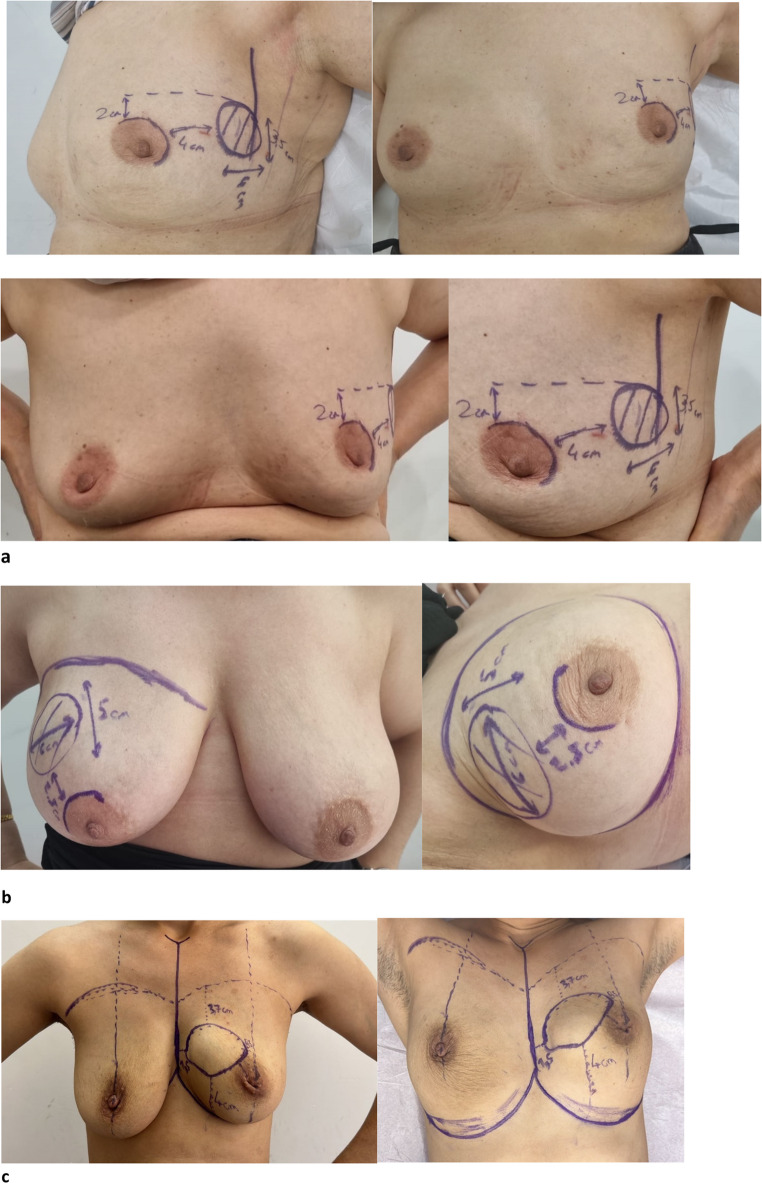
**a, b,c** – Topographic sketching examples

In the TS group, tumor borders were identified based on detailed physical examination and imaging prior to NAC. The location, distance to the areola, and projection on the skin were marked and photographed. These records were archived in the patient’s file. After NAC, the tumor response and changes in location were evaluated using post-treatment imaging and clinical findings. Prior to surgery, the topographic sketch was redrawn on the skin based on archived and current data, and the marked area was excised without the use of clips or wires (Fig. 2.a, b,c).

Frozen section analysis was performed intraoperatively in all patients to assess surgical margins. Whenever a margin concern was identified during intraoperative assessment, an appropriate re-excision was performed during the same surgical session. The following variables were recorded and compared between the two groups: clinicopathological features, margin status, pathological response, excised tissue volumes, reoperation rates, and local recurrence.

### Statistical analysis

The normality of continuous variables was assessed using the Shapiro–Wilk test. Categorical variables were expressed as frequencies and percentages, while continuous variables were presented as median (range), mean, and standard deviation. Group comparisons for continuous variables were performed using the Mann–Whitney U test. For categorical data, Pearson’s Chi-square, Continuity Correction, or Fisher’s Exact Test were used as appropriate. A p-value < 0.05 was considered statistically significant. All analyses were conducted using SPSS software version 25 (IBM Corp., Armonk, NY, USA).

## Results

The mean age of the patients was 48 years, with a mean of 47 years in the TS group and 49 years in the RM group. Among the 208 patients, 187 were classified as clinical T1–T2, and 21 as T3–T4. Axillary involvement was present in 195 patients prior to NAC, while 13 patients had no nodal involvement.

A total of 168 patients had unifocal tumors and 40 had multifocal tumors. Post-NAC, 91 patients exhibited a pathological complete response (pCR) in the breast, while 117 had a partial response. Regarding molecular subtypes, 13 patients had luminal A, 108 luminal B, 34 HER2-positive, and 53 triple-negative tumors. No statistically significant differences were observed between the TS and RM groups in terms of age, T stage, N stage, multifocality, pathological response, or molecular subtypes (Table 1).

**Table 1:** Patient and tumor characteristics

Variables		TS (*n* = 106)	RM (*n* = 102)	Total (*n* = 208)	
	Category	*n*(%)	*n*(%)	*n*(%)	*P*-value
Age, mean ± SD	All	47.18 ± 11.16	49.14 ± 10.76	48.14 ± 10.98	0.199^a^
cT Stage	cT1-2	93(87.7)	94(92.2)	187(89.9)	0.408^c^
	cT3-4	13(12.3)	8(7.8)	21(10.1)	
cN Stage	cN0	9(8.5)	4(3.9)	13(6.3)	0.283^c^
	cN+	97(91.5)	98(96.1)	195(93.8)	
Multifocality	positive	21(19.8)	19(18.6)	40(19.2)	0.968^c^
	negative	85(80.2)	83(81.4)	168(80.8)	
pCR (breast)	positive	49(46.2)	54(52.9)	103(49.5)	0.333^b^
	negative	57(53.8)	48(47.1)	105(50.5)	
pCR(axilla)	positive	74(69.8)	66(64.7)	140(67.3)	0.433^b^
	negative	32(30.2)	36(35.3)	68(32.7)	
pCR (breast+axilla)	positive	42(39.6)	49(48)	91(43.8)	0.221^b^
	negative	64(60.4)	53(52)	117(56.3)	
Molecular subtype	luminal-A	6(5.7)	7(6.9)	13(6.3)	0.600^b^
	luminal-B	33(31.1)	30(29.4)	63(30.3)	
	luminal Her2(+)	22(20.8)	23(22.5)	45(21.6)	
	non-luminal Her2(+)	14(13.2)	20(19.6)	34(16.3)	
	TN	31(29.2)	22(21.6)	53(25.5)	
Specimen volume (cm3), median (IQR)	pCR	216(99–411)	162(78–274)	196(92–349)	0.136^e^
	non-pCR	140(73–280)	185(85–289)	168(74–280)	0.477^e^
	total	174(78–348)	168(84–275)	172(80–308)	0.611^e^
Reoperation	excision	1(0.9)	0(0)	1(0.5)	0.999^d^
	mastectomy	0(0)	1(1)	1(0.5)	0.490^d^
Local recurrence	Yes	3(2.8)	3(2.9)	6(2.9)	0.999^d^
	No	103(97.2)	99(97.1)	202(97.1)	
Follow-up time (months)	median (IQR)	60(48–96)	60(48–72)	60(48–84)	0.084^e^

p > 0.05 a: Independent sample t test, b*:* Pearson chi-square test, c*:* Continuity correction test, d*:* Fisher’s exact test, e*:* Mann-whitney U test, *SD *Standard deviation, *TS *Topographic sketching, *RM* Radiologic marking, *IQR(PP*_25_-P_75_) Inter Quantile Range

Only two patients required reoperation due to positive surgical margins: one underwent re-excision in the TS group, and one underwent mastectomy in the RM group.

There was no significant difference in excised tissue volumes between the two groups. The median excision volume was 174 cm³ in the TS group and 168 cm³ in the RM group.

During a median follow-up period of 60 months, local recurrence occurred in 6 patients—3 from each group. Three patients with recurrence had non-luminal tumors.

## Discussion

Breast-conserving surgery (BCS) offers superior cosmetic, psychological, and sexual outcomes compared to mastectomy [12]. Therefore, BCS should be prioritized in all eligible breast cancer patients. However, in tumors that become non-palpable following neoadjuvant chemotherapy (NAC), accurate identification of the excision area can be challenging, potentially increasing the risk of positive surgical margins [13]. To address this, pre-treatment tumor localization is widely recommended [14, 15].

Radiopaque clip placement is a standard technique used to facilitate wire-guided excision in patients showing a complete or near-complete response to NAC. Multiple studies have confirmed the reliability and effectiveness of this method. Edeiken et al. reported that in 47% of patients with a complete or near-complete response, radiopaque clips were the only identifiable markers for tumor bed localization [16]. Likewise, Oh et al. observed a lower local recurrence rate at five years in 373 patients who received clip marking prior to NAC and recommended its routine use in candidates for breast-conserving surgery [17].

In our study, local recurrence was observed in two patients in each group (a total of 4 patients, 2.5%). The local recurrence rates were similar between the two groups at two-year follow-up.

Successful application of clip and wire localization requires a high level of coordination between surgical and radiological teams. These methods are invasive and associated with potential complications such as pain, hematoma, and wire migration. In contrast, topographic sketching (TS) is a non-invasive, cost-effective technique that eliminates these risks. It is easily applicable in breast surgery units that maintain a functional archiving system. Regardless of the method used, each case should be evaluated by a multidisciplinary team to plan the most appropriate surgical approach.

Excised breast volume and the need for reoperation are two key factors that significantly impact cosmetic outcomes following BCS [18, 19]. In our study, there were no significant differences in resected volumes or reoperation rates between the TS and RM groups. The routine use of intraoperative margin assessment may have contributed to the low reoperation rates observed in our study. Whenever a potential margin issue was identified during intraoperative pathological evaluation, appropriate re-excision was routinely performed during the same surgical session. We are aware that frozen section analysis, although useful in selected patients, is not always effective and may be time-consuming. In particular, it is not consistently reliable in detecting ductal carcinoma in situ at the surgical margins. One potential advantage in our setting is that margin assessments were performed by a pathologist with specific expertise in breast pathology, which may have contributed to higher diagnostic accuracy.

Skin tattooing has also been explored as an alternative to radiological localization techniques [20, 21]. In this method, the tumor borders are marked on the skin with permanent ink prior to NAC, and no clip or wire placement is needed. Bravo et al. compared skin tattooing with radiological localization in 149 patients and found no significant difference in margin positivity or the need for reoperation, although excised volumes were higher in the tattoo group [22]. Similarly, Lannin et al. reported a 91% success rate in achieving negative margins using the skin tattooing method [23]. In their study comparing clip localization with skin tattooing, Kut et al. demonstrated that the tattooing method could be a viable alternative to clip-based localization [24].

An additional advantage of TS is its ability to document the original tumor borders at initial presentation. Since tumor regression after NAC may occur in a concentric manner or present as scattered residual foci (“Swiss cheese” pattern), awareness of the pre-treatment tumor extent may help ensure complete removal of all disease.

It is important to consider that the effectiveness of topographic sketching may be limited in patients with large and ptotic breast anatomy. Notably, such anatomical features can also increase the technical difficulty of surgery in the radiological marking (RM) group.

The primary limitations of the TS method are its dependency on pre-NAC documentation and institutional infrastructure. TS cannot be applied to patients who were initially evaluated at other centers or who lack archived images. Loss of stored photographs may also hinder implementation. The success of this technique relies on robust archiving systems and strong interdepartmental coordination. At our institution, an online archive accessible to all healthcare personnel ensures that pre-treatment breast photographs are routinely obtained and stored for all newly diagnosed patients.

## Conclusion

Excised breast volumes, reoperation rates, and local recurrence rates were comparable between the radiological marking and topographic sketching groups. Topographic sketching appears to be a promising, non-invasive, cost-effective, and easy-to-implement alternative that provides valuable pre-treatment tumor localization data without procedural complications.

## Data Availability

The datasets analysed during the current study are available from the corresponding author on reasonable request.
